# A review of retention mechanism studies for packed column supercritical fluid chromatography

**DOI:** 10.1002/ansa.202000144

**Published:** 2021-01-21

**Authors:** Le Si‐Hung, Takeshi Bamba

**Affiliations:** ^1^ Division of Metabolomics, Medical Institute of Bioregulation Kyushu University Fukuoka Japan

**Keywords:** Packed column supercritical fluid chromatography, Retention behavior, Retention mechanism, Supercritical fluid chromatography

## Abstract

The packed column supercritical fluid chromatography has risen as a promising alternative separation technique to the conventional liquid chromatography and gas chromatography. Although the packed column supercritical fluid chromatography has many advantages compared to other chromatographic techniques, its separation mechanism is not fully understood due to the complex combination effects of many chromatographic parameters on separation quality and the lacking of global strategies for studying separation mechanisms. This review aims to provide recent information regarding the chromatographic behaviors and the effects of the parameters on the separation, discuss the results, and point out the remaining bottlenecks in the packed column supercritical fluid chromatography retention mechanism studies.

**Abbreviations**
cSFC: open‐tubular capillary supercritical fluid chromatography
EM: empirical approaches
ETM: extra thermodynamic approaches
LSER: linear solvation energy relationships
MeO: methoxyl
MeOH: methanol
NH_4_Ac: ammonium acetate
OH^–^
: hydroxyl
pSFC: packed column supercritical fluid chromatography
QSRR: quantitative structure‐retention relationships
scCO_2_
: supercritical CO_2_
SFC: supercritical fluid chromatography
TM: thermodynamic approaches
UC: unified chromatography

## 1 INTRODUCTION

Supercritical fluid chromatography (SFC) is a chromatographic separation technique, which employs a supercritical fluid mixed with small amounts of modifiers and additives as the mobile phase.^1^ Since the first introduction in 1962, SFC using both open‐tubular capillary (cSFC) and packed columns (pSFC) has been considered an alternate (or extension) for normal phase liquid chromatography (NPLC) and gas chromatography (GC).^2^, ^3^, ^4^, ^5^, ^6^ When the temperature and the pressure of a gas approach its critical point condition, the gas will stay in a homogeneous state; hence, a supercritical fluid starts to emerge.^7^ The density, viscosity, and diffusion coefficients of supercritical fluids can change continuously from values of gas to values of liquid, without observation of phase changing. At practical pressures for applications (5‐30 MPa), supercritical and subcritical mobile phases have lower viscosity and higher diffusivity relative to liquid mobile phases, which makes SFC separation faster and more efficient in comparison with LC separation.^6^, ^8^, ^9^ Over time, cSFC has slowly lost its popularity, while pSFC applying common high‐performance liquid chromatography (HPLC) columns has taken over most applications.^3^ The main reasons for the popularity of pSFC are larger sample loading capacity, higher analytical speed, better detection limits, and a wider range of compounds that can be analyzed.^10^ Other reasons are the ability to use co‐solvents, additives, gradient elution, and the wide availability of commercial LC‐dedicated columns.^5^

**FIGURE 1:**
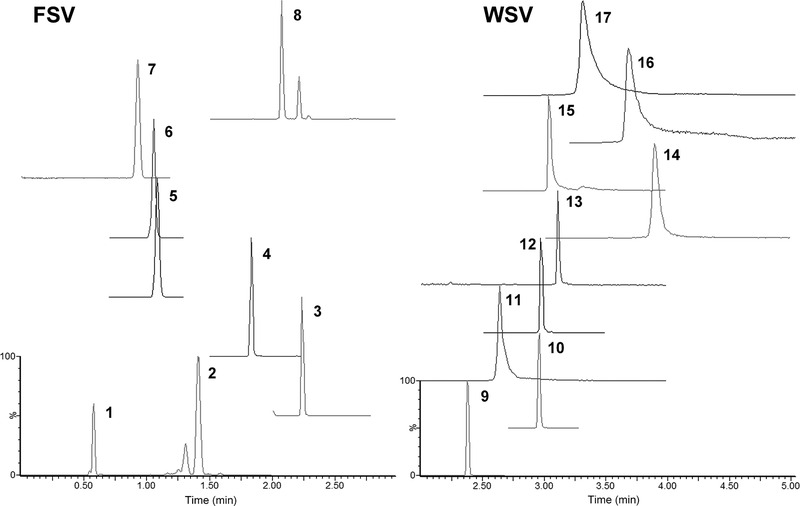
MRM chromatograms of 17 water‐ and fat‐soluble vitamins, for example,(1) A acetate, (2) A palmitate, (3) D2, (4) α‐tocopherol, (5) K2, (6) K1, (7) α‐tocopherol acetate, (8) β‐carotene, (9) nicotinamide, (10) Nicotinic acid, (11) Pyridoxine, (12) d‐pantothenic acid, (13) Biotin, (14) Thiamine, (15) Riboflavin, (16) B12, and (17) VC, using unified chromatography method. Reprinted from^18^ with permission

Despite high expectations, in the past SFC faced many challenges regarding instrument design, repeatability, and robustness that limited its applications in the petrochemical industry for the separation of nonpolar, high molecular weight, thermally labile, and non‐volatile molecules.^5^, ^11^, ^12^ Since 2010, many improvements have been developed for new generation SFC instruments (e.g. new cooling SFC pumps, new automatic backpressure regulator based on ultra‐high performance liquid chromatography technology, higher upper‐pressure limits, lower void volumes, and full compatibility to modern packed columns), which allow SFC‐based methods to improve not only the repeatability and reproducibility but also sensitivity and selectivity.^3^, ^5^, ^13^, ^14^ Compared to NPLC, in SFC column equilibration requires less time, and trace of water doesn't affect analyte retention time.^9^ Moreover, in terms of chromatographic column's kinetic performance, the lower viscosity of supercritical CO_2_ (scCO_2_) and the higher analyte diffusion in supercritical fluids result in better mass transfer rate, higher optimum mobile phase velocity with a low pressure drop, and greater column efficiency.^15^ Furthermore, using smaller particle sizes reduces diffusion path lengths of analyte inside the column and improves the mass transfers of analyte between the mobile phase and the stationary phase. This results in less band broadening, hence better chromatographic peak resolution. Wang *et al*. analyzed 3′‐demethyl‐NOB and 4′‐demethyl‐NOB metabolites on several stationary phases using both SFC and LC.^16^ While reversed‐phase chromatography (RPLC) was unable to separate these metabolites, NPLC and SFC were capable to separate the metabolites on the diol, pyridine, and silica stationary phases. Moreover, by using modifiers (e.g. alcohols) and additives (e.g. salts and waters), a wider analyte polarity range can be simultaneously analyzed by SFC.^17^ For instance, Taguchi *et al*. demonstrated an SFC‐MS/MS method for the simultaneous analysis of compounds with log*P_ow_* values ranges from −2.1 to 10.1 in just one run using a single C18 column.^18^ In the method, the SFC gradient was changed continuously from pure CO_2_ to pure methanol (MeOH). Hence, the mobile phase state was changed from supercritical to subcritical and finally to a liquid without a discontinuous phase transition. The proposed method could separate all analytes in less than 7 min, with good reproducibility in terms of retention time and peak area, even for some early eluting compounds (Figure 1).

**FIGURE 2:**
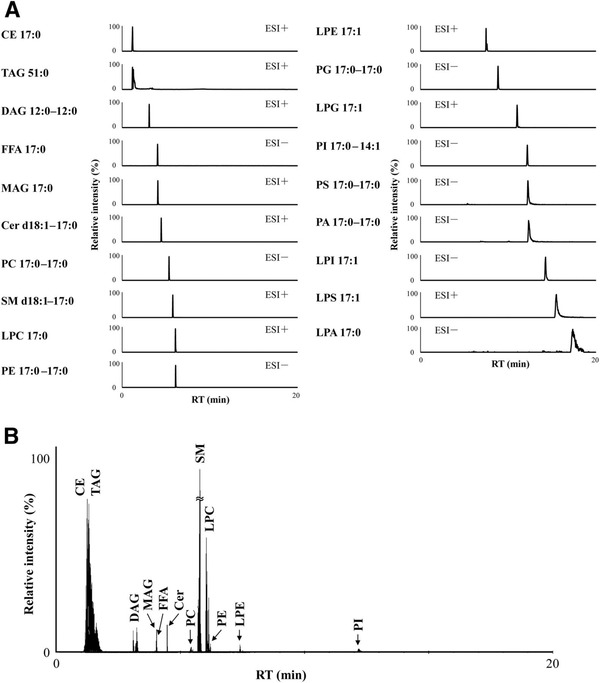
High‐resolution SFC separation of each lipid class using a DEA column. (A) MRM chromatograms of 19 lipid classes internal standard mixture. (B) MRM chromatograms of lipids in the rabbit plasma. Reprinted from^26^ with permission

The employing of such gradient was designated as unified chromatography (UC), proved the possibility of unified fluid chromatography concept, where different chromatographic modes could be carried out in a series of a single chromatographic run.^17^, ^19^ By completely removing CO_2_ and using suitable stationary phases, the working polarity range of the UC method was significantly improved. Moreover, it crossed a “thin‐red‐line” between SFC and LC and finally “unified” the two chromatographic techniques. The coupling of columns with different polarity ranges in SFC is much easier due to the same mobile phase can be applied.^3^, ^20^ Compared to GC, SFC operates at lower temperatures, has a larger molecular weight working range, and comparative column efficiencies. Also, the selectivity of SFC‐based methods can be tuned via controlling column pressure and column temperature.^6^ SFC combined with mass spectrometry (MS) has extended its application to many new research fields including bio‐analysis, metabolomics, drug, plant, food, and environmental analysis.^21^, ^22^, ^23^, ^24^, ^25^, ^26^, ^27^, ^28^, ^29^, ^30^, ^31^ For instance, Ishibashi *et al* reported a high throughput simultaneous analysis of more than 400 pesticides in food samples using SFC combined with high‐resolution mass spectrometry (HRMS).^32^ Takeda *et al* proposed a widely‐targeted quantitative lipidomics method using SFC‐MS/MS for the simultaneous analysis of 19 lipid classes in biological samples (Figure 2).^26^

Although a direct and fair comparison regarding the sensitivity between analysis methods is challenging, many studies showed comparable sensitivities between SFC/MS methods and LC/MS methods.^3^, ^33^ For instance, using a set of more than 400 pesticides covering a wide polarity range, Fujito *et al* found a significantly higher sensitivity could be obtained by SFC/MS compared to that obtained by LC/MS.^33^ Using the new generation SFC instruments, many studies also confirmed that SFC‐based methods had good repeatability and reproducibility compared to other methods.^34^ Losacco *et al* investigated retention time variability of an anti‐doping analysis method using SFC/MS under reproducible conditions.^35^ In this investigation, a set of 51 doping standards was spiked in diluted urine samples and measured over 4 months on 12 columns of three stationary phases (e.g. Acquity Torus 2‐Picolylamine, Viridis BEH, and Acquity HSS C18 SB). Results showed that two of the studied columns had excellent retention time repeatability with a relative standard deviation (RSD) value of less than 2%. Moreover, a good inter‐batch reproducibility could be achieved from the same stationary phase of different production lots for all columns.

**FIGURE 3:**
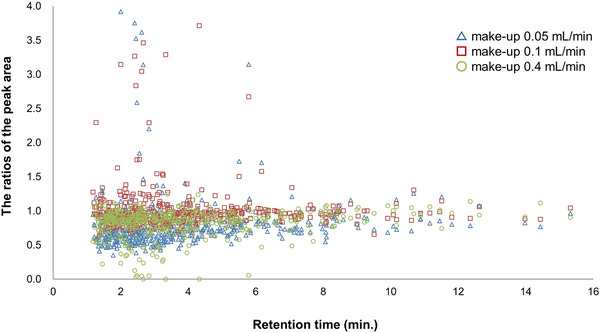
The comparison between the normalized signal intensity of 441 compounds under three flow rates of make‐up solvent using the ratio of the peak area for 0.05, 0.1, and 0.4 mL/min to that of 0.2 mL/min in SFC/MS. The ratio of the peak area of each compound was plotted in order of elution. Reprinted from^33^ with permission

Despite the advantages, SFC still faces many difficulties in contrast to other separation techniques. For example, the SFC pump head's temperature needs to be kept at low temperatures (e.g. ‐5°C) to pump CO_2_ in the liquid state.^36^ Besides, SFC systems often have higher dead volumes compared to LC systems due to their more complex instruments.^3^ Additionally, contamination may happen when supercritical fluid‐grade and research‐grade CO_2_ are packed into small containers, consequently causing high MS background noise.^6^ A makeup solvent is added to the SFC mobile phase to prevent compound precipitation when CO_2_ evaporates under atmospheric conditions or to enhance MS response signal.^3^, ^37^, ^38^ However, the choice of makeup solvent and makeup flow rate needs to be considered carefully, since they can cause the MS signal suppression. Figure 3 shows the effects of makeup solvent flow rate on MS signal for analysis of 400 pesticides using SFC‐MS/MS.

Furthermore, compressibility and non‐ideal behaviors of supercritical fluids create simultaneous velocity, temperature, pressure, density, and retention factor gradients along the column, hence affecting the column efficiency.^39^, ^40^ SFC also has a problem regarding poor reproducibility of early eluting compounds, which is caused by a large volume of injection or high solvent strength of the sample solvent compared to the mobile phase.^15^, ^41^ This problem can be avoided by carefully choosing the injection volume (less than 0.5% of the column sample volume is recommended) or carefully dissolving analyzed sample in suitable solvents (e.g. acetonitrile, mixture of 30% tetrahydrofuran/70% heptane (v:v)). Nevertheless, too large injection volume, which can cause distortion of analyte peak, is the main disadvantage of SFC.^42^, ^43^ Although adding modifiers extends the working polarity range of SFC, RPLC is preferred for the analysis of highly polar compounds.^9^

One of the most important contributions of SFC to separation science is the possibility of developing a universal chromatography technique that merges GC and LC theories; hence, finally, at least one true theory of chromatography can be found.^10^ SFC has been widely applied as a “green”, high efficiency, a hybrid chromatographic technique to challenge remained bottlenecks in many applications, especially in metabolomics.^1^, ^3^

## 2 PACKED COLUMN SUPERCRITICAL FLUID CHROMATOGRAPHY

pSFC is a chromatographic separation technique employing supercritical mobile phases and packed columns, has the following distinctive characteristics^44^:

‐CO_2_ is the most popular used supercritical fluid in pSFC due to its low critical parameters (31.1°C and 7.36 MPa), non‐flammable, low cost, and non‐toxicity.
‐The employed mobile phase, usually a binary or ternary mixture with CO_2_, is delivered by a regular high‐pressure pump system equipped with a cooling system.^6^
‐The supercritical conditions are maintained by a backpressure regulator (BPR) installed post‐column to control column backpressure.^45^
‐The columns are operated at temperatures near the critical temperature of the fluid.
‐A wide polarity range of stationary phases can be routinely used.
‐Both atmospheric pressure chemical ionization and electrospray ionization are applied in pSFC/MS.^33^, ^46^ However, for the electrospray ionization, a polar modifier is usually added to the mobile phase to aid the ion production especially when using the gradient of the mobile phase compositions.^3^

pSFC has many practical advantages relative to other techniques such as shorter analysis time, higher throughput, faster equilibration, and shorter cycle time. In 2020, Konya *et al* reported a rapid and simultaneous method for direct analysis of 100 cationic and amphoteric compounds (log*P*_ow_ values from –5.9 to 1.7) within 10 min using pSFC and a mobile phase of CO_2_/MeOH/water/TFA with a ratio of 70/27/3/0.15 (v/v/v/v).^47^ pSFC has benefited considerably from revolutions of LC‐dedicated columns, although their separation conditions are quite different.^39^ Almost the same instrument and software developed for HPLC can be used in pSFC. Operational cost per sample of pSFC is lower due to less organic solvent is consumed, and analytes can be enriched in a quite small solvent volume. Lastly, adjusting both mobile phase compositions and column conditions can change analyte solubility, and hence promotes a wider range of interactions between analytes and stationary phase, which in turn offers more flexibility to tune the selectivity in pSFC.^39^

### 2.1 Carbon dioxide‐based mobile phase

The most used mobile phase in pSFC is CO_2_ due to its easy reachable critical parameters, low cost, nontoxicity, and non‐inflammability.^5^ Due to low dielectric constant, zero molecular dipole moment, and high quadrupole moment of CO_2_, it is considered a nonpolar, quadrupole solvent, an alternative for hydrocarbon solvents, and its polarity is considered being similar to hexane or heptane. Nevertheless, many compounds containing hydrocarbon chains are not highly soluble in CO_2_. Whereas several fluorocarbons, poly(ether‐carbonate) co‐polymers, and sugar acetates are considered CO_2_‐philes. Recent studies suggested that CO_2_ could behave like both weak Lewis acid and Lewis base and took part in conventional or nonconventional hydrogen‐bonding interactions.^6^, ^48^, ^49^, ^50^, ^51^, ^52^ Strubinger *et al* investigated surface excess adsorption isotherms of supercritical and subcritical CO_2_ on various stationary phases.^53^ Results from the study showed that maximum multilayer adsorption on stationary phase was obtained at condition near CO_2_ critical condition. It proved that the stationary phase adsorbed at least a monolayer of condensed mobile phase under common practical SFC conditions. Due to scCO_2_ has weak eluting strength, most current SFC studies employ gradient elution with organic modifiers composition and polar additives at low concentration to improve the retention and peak shape of analytes of interest.^18^, ^39^, ^54^

The modifier, mostly an alcohol, is the second component used in pSFC mobile phase.^55^ These modifiers can modify polarity, critical temperature, and critical pressure of the neat CO_2_ mobile phase.^56^ Adding a small amount of miscible polar modifier to scCO_2_ mobile phase increases the solvating power of the mobile phase, which leads to better dissolving of polar compounds.^6^ Solubilities of almost polar organic compounds (e.g. alcohols, ethers, chloroform, and dimethyl sulfoxide) are better than that of CO_2_, and thus they can be used as modifiers in pSFC. It is a common thought that adding modifiers may change the density of pSFC mobile phase. However, results from Tarafder's work indicated that the effect of adding modifiers might be insignificant. The addition of modifiers increases the viscosity of the mobile phase, and hence reduces the mobile phase compressibility, which in turn increases pressure drop along the column but does not significantly change the total density of the mobile phase.^57^ The addition of modifiers also affects the volume, apparent pH, polarity, and solvating power of the mobile phase. For example, West *et al*. confirmed that the apparent pH (^s^_w_pH) of CO_2_ mobile phase with MeOH was approximately 5, and ^s^_w_pH of CO_2_ mobile phase with MeOH and acidic additives (or water) was less than 1.7.^58^ The mobile phase became more acidic when increasing MeOH percentage due to methoxylcarbonic acid formation.^3^ However, when adding basic additives to the mobile phase an insignificant change of ^s^_w_pH was observed. Possible reasons for this insignificant change can be the titration by alkoxylcarbonic acid or the ion pairing formed with alkoxycarbonate. There is evidence of strong adsorption of modifiers on the surface of the stationary phase, and thus polarity, volume, and three‐dimensional structure of the stationary phase are changed. Furthermore, the adsorbed modifiers cover active sites on the stationary phase surface, which prevent nonspecific interactions with analytes and function as a part of the stationary phase.^39^, ^44^, ^54^, ^59^, ^60^, ^61^, ^62^ Regarding effects on the analyte retention, based on an investigation of more than 100 compounds using different stationary phases, West *et al* concluded that some columns were more affected by solvent nature than the others were.^61^ Results from the study also showed that although compound retentions and separation processes depended strongly on the nature of the stationary phases, modifiers also contributed. Zou *et al* found that the addition of modifiers considerably increased apparent column efficiency for extremely retained nonpolar compounds used in this study on some columns.^63^ Whereas, for polar compounds in the study, improvement of apparent column efficiency was related to the amount and nature of the modifiers. Moreover, the composition of pSFC mobile phase has more impact on the analyte retention than the density of the mobile phase.^6^, ^64^ However, when the less polar modifier is used, the effects of the density of the mobile phase becomes more pronounced.^15^ MeOH is one of the strongest solvents that dissolve fully in CO_2_ and one of the most common modifiers used in pSFC. Adding a small amount of MeOH to the mobile phase can greatly improve the mobile phase solvent strength, which relates to the ability of a solvent to elute analytes more quickly from the column. Variation of the modifier composition causes the changes in the mobile phase solvation strength, and variation of the modifier concentration is the main reason causing the changes of the analyte retention.^10^, ^65^ In most cases, increasing the modifier fraction reduces the analyte retention; however, if too much of the modifier is used, super(sub)critical conditions of the mobile phase cannot be maintained.^66^

**FIGURE 4:**
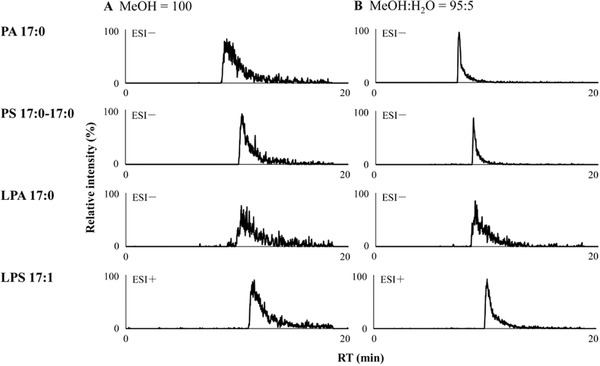
The peak shape improvement of four polar lipids by adding 5% water to the modifier solvent. (A) Methanol with 0.1% (w/v) ammonium acetate. (B) Methanol/water (95/5, v/v) with 0.1% (w/v) ammonium acetate. Reprinted from^26^ with permission

Nevertheless, for analysis of strong acids, bases, and some polar compounds adding modifiers is not always enough. Therefore, the third component in pSFC mobile phase, additives, is further used at low concentrations to improve the retention and peak shape of the analytes.^10^ Common additives used in pSFC are usually very polar compounds such as organic acids, organic bases, amines, and volatile salts.^18^ As a rule of thumb, the acidic additives help the analysis of acidic compounds, and the basic additives benefit the analysis of basic compounds. Water is used as a very polar additive together with other polar organic modifiers to improve the retention and peak shape of very polar compounds.^34^, ^67^ Figure 4 shows an example regarding peak shape improvement when water was added to pSFC mobile phase consisting of CO_2_ and MeOH (Figure 4).

Regarding MS coupling, both the acidic and basic additives often cause the ionization suppression, while adding volatile salts as additives can improve chromatographic separation for both acidic and basic compounds without suppressing ionization. The addition of additives can affect the density, polarity, and solvating power of the supercritical mobile phase. However, the effects of additives addition on the density of the mobile phase are relatively small due to the small amount of additives is often used (less than 2% w/v). In contrast, the effects on the polarity of the mobile phase are significant due to a big difference in the polarity between highly polar additives and other components in the mobile phase. Additives are also adsorbed on pSFC stationary phases, contributing effects to the separation processes. Similar to the adsorbed modifier molecules, the adsorbed additive molecules can cover, interact with active sites on the surface of the stationary phase, and function as parts of the stationary phase. Hence, these adsorbed molecules change the polarity, physical thickness, and chemical nature of the stationary phase. Regarding the effects of additives on retention factors of analytes, Cazenave‐Gassiot *et al* showed that increasing concentrations of ammonium acetate (NH_4_Ac) from 0 to 60 mM greatly shortened retention of seven aromatic drugs and improved peak shapes of the analytes on both endcapped and non‐endcapped 2‐ethyl‐pyridine (2‐EP) columns.^68^ Addition of a small amounts of water as the additive was found to improve column efficiency and retention factor for most compounds used in the study on some pSFC stationary phases including FructoShell‐N phase.^54^ Other common additives are amines, frequently used to analyze basic analytes, although the primary and secondary amines can interact with CO_2_‐alcohol mixtures.^67^ Glenne *et al* showed that the fraction of amines highly affected column efficiency and peak shape of basic pharmaceuticals used in the study under SFC conditions.^69^ In 2019, Liu *et al* reported the effect of bicarbonate as a chaotropic agent that disrupted undesired H‐bonding interactions in separations of cyclic peptides using SFC when ammonium was used in water‐rich MeOH modifiers.^70^ It was found that compared to traditional basic additives, using ammonium hydroxide in water‐rich MeOH modifiers resulted in a better performance in terms of the peak shape, analyte retention, selectivity, and ionization efficiency. The results from West *et al* showed that on the hybrid silica stationary phase, increasing NH_4_Ac concentrations (0‐25 mM) increased the retention of acidic compounds, while only moderated effects on the retentions of basic and neutral compounds were observed.^55^ Nevertheless, poor reproducibility was observed when small amounts of additives appeared in the sample solvent. Raimbault *et al* reported that contradictory to previous statements regarding the additive effects, the highly concentrated additives showed negligible effect on the mobile phase polarity.^71^ Conversely, the additive effects on the mobile phase acidity was significant, and thus causing the change in the ionization state of carboxylic acid function of the analyte. The authors also reported that at high concentrations of isopropylamine (as a basic additive), the adsorption of additives on the stationary phase showed the highest impact on retention mechanisms. However, the addition of additives also has many disadvantages such as increasing noise for low‐wavelength absorbance detection, corroding silica‐based stationary phases, introducing ionization interference, and causing unwanted reactions with analytes.^39^ Additionally, the addition of additives can suppress the ionization of analytes when coupling with MS, form ion pairs with ionic compounds, and change the solubility of the analyte in the mobile phase.

#### 2.1.1 Effects of pressure and temperature

**FIGURE 5:**
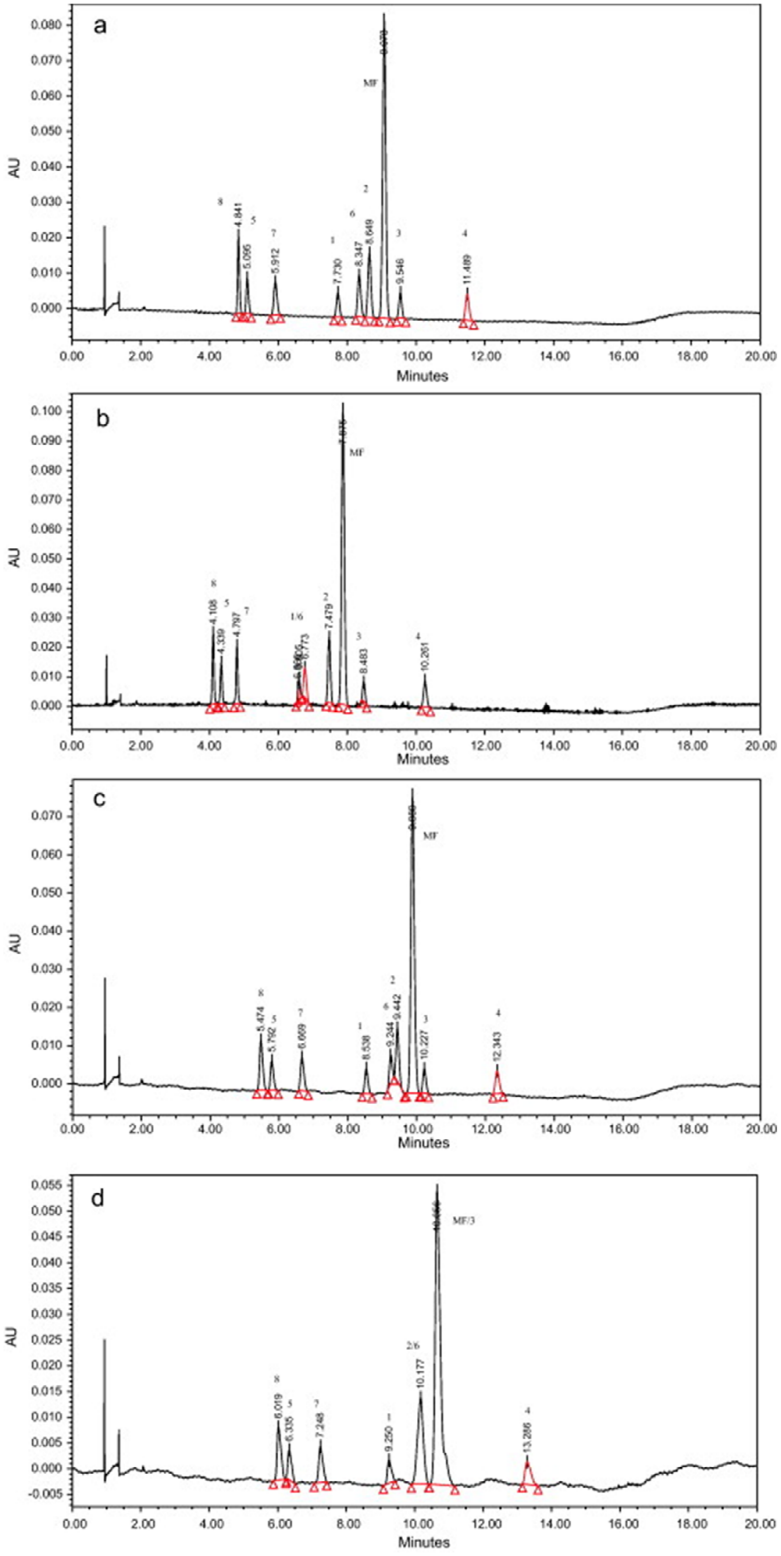
Effect of temperature and pressure of the mobile phase on retention and selectivity of nine drugs using a silica column. Reprinted from^65^ with permission

In comparison with typical liquids, the properties of supercritical fluids are greatly influenced by chromatographic conditions such as the column temperature, column pressure, particle size, packing density, and column length. At too low pressure, or too high temperature, or too high modifier concentration, both vapor and liquid states of the mobile phase can co‐exist inside the column, which leads to impossible chromatographic separations.^6^ To avoid this situation, the pressure needs to be maintained above the two‐phase region at the temperature used. Additionally, due to the pressure drop, the supercritical mobile phase expands along the column, which leads to a decrease in the column temperature, and hence generating large axial and radial gradients of the viscosity, density, and temperature. These gradients forming a non‐uniform radial distribution of compound migration velocities that may affect directly solute band profiles and cause significant column efficiency losses.^72^, ^73^ Furthermore, it was found that solute heat adsorption on various stationary phases showed a high impact on the analyte retention.^74^, ^75^ Using kinetic plots that provide information regarding the shortest analysis time to obtain a specific efficiency or the maximum efficiency can be obtained with the system in a given time, Delahaye *et al* confirmed that the backpressure, column temperature, and modifier amount showed strong effects on the SFC separation.^76^ The authors concluded that it was highly recommended to operate SFC at adequate average pressure and backpressure for preventing the efficiency losses.^77^ The changes in the mobile phase density caused by the variations of temperature or pressure can influence both the analyte retention and the selectivity in SFC.^20^ Many studies pointed out the relationship between the retention factor, mobile phase density, and analyte solubility.^65^, ^78^, ^79^, ^80^, ^81^, ^82^ In short, the variation of the mobile phase density changes the mobile phase solvation power, which leads to varying analyte solubility, hence affecting the analyte retention time. Wang *et al* evaluated effects of the column temperatures (30‐40°C) on the retention time of nine drugs using a silica stationary phase under the constant pressure of 10 MPa.^65^ The results showed that increasing the column temperature increased the analyte retention, while slightly decreased the compound selectivity (Figure 5). Moreover, the effect of increasing temperature on the analyte retention was more significant at higher temperatures indicated that decreasing the mobile phase density at these temperatures might be a reason.

Ovchinnikov *et al* evaluated effects of temperatures (25‐55°C) and pressures (11‐18 MPa) on the analyte retention and the selectivity of 89 compounds from various chemical classes using four polar stationary phases (e.g. ethylene‐bridged hybrid silica, cyanopropyl, 2‐ethylpyridine, and zwitterionic sulfobetaine).^83^ It was found that only a moderate effect on the selectivity was observed, and increasing the pressure at constant temperatures increased the mobile phase density and its eluting power, hence decreasing the retention of all compounds. However, the authors concluded that the effect of the temperature on the analyte retention depended on both the compounds and the stationary phases. Losacco and Fekete investigated the influence of untraditional column temperatures on the retention of several compounds eluted in different percentages of modifiers using SFC.^84^ The results from the study indicated that when low percentages of the modifiers were used, the use of low temperatures resulted in better column performance; however, when higher percentages of modifiers were used, the use of high temperatures got more advantages. Additionally, the isomeric separation of *E/Z*‐endoxifen showed higher peak resolution when applying low temperature (–5°C). The improvement of the resolution could be explained by the fact that at the low temperature the available energy in the system and the rotation phenomenon were reduced, and thus obtaining better stability for these isomers. A study from Vajda *et al* indicated that increasing the temperature while decreasing the backpressure increased the thickness of the absorbed organic solvent layer above the silica gel surface under the SFC conditions.^85^

To sum up, at a constant temperature, increasing the pressure will increase the density and the solvating power of the mobile phase, and thus decreasing the retention factor.^6^, ^10^, ^86^ At higher temperatures near the critical temperature of the mobile phase, the retention factors of analytes change quickly when the pressure changes due to rapid variation of the mobile phase density under these conditions. At further higher temperatures than the critical temperature, increasing the pressure shows an insignificant effect on the analyte retention. In contrast, at constant pressure, the relationship between the retention factor and the temperature is complicated. Depending on the nature of the analyte, the retention factor can either decrease or increase when the temperature increases.^86^ Varying temperature changes the mobile phase density and kinetic energy of solute, hence changing the retention, the selectivity of compounds of interest. At low pressure, the solvating power of the mobile phase decreases when the temperature increases, which may be caused by the intense variation of the mobile phase density happened at these conditions; however, at high pressure, the solvating power increases with the increasing of the temperature. Additionally, the temperature and pressure can affect the diffusion and the viscosity of the mobile phase. Hence, they can contribute more impact to the efficiency, analyte retention, and selectivity especially when using pure CO_2_, or low modifier concentrations, or low outlet pressures.

### 2.2 Stationary phases in pSFC

Introduced during the 2000s, 2‐ethylpyridine (2‐EP) was the first and the most famous SFC‐dedicated stationary phase, especially for analysis of basic compounds.^87^ After that, other stationary phases such as 4‐ethylpyridine, pyridine amide, aminophenyl, 2‐picolylamine, poly(4‐vinylpyridine), and 1‐aminoanthracene were subsequently commercialized.^5^, ^87^ Moreover, all existing LC‐dedicated stationary phases can be used in pSFC including the highly polar stationary phases (e.g. silica), the less polar stationary phases (e.g. C_18_ or ODS bonded silica), and the mixed‐polarity stationary phases.^88^ However, due to the high diffusivity of the supercritical mobile phase, the surface silanols, including free, geminal, and associated silanols, become more problematic as they can affect the retention behavior of analytes. Regarding the peak shape of polar analytes, the free‐silanols, which are more acidic than the rest of silanols, may cause peak tailing, while the other silanols may result in more narrow peaks.^54^ There is evidence that under the practical SFC conditions the residual silanols can react with the alcohol modifiers to form silyl ether, hence decreasing the stationary phase hydrophilicity.^89^ Nevertheless, the reaction is reversible with the appearance of water.^6^ Additionally, the non‐homogeneity of the silica surface, which can cause non‐homogeneous adsorption and multilayer formation of water, may lead to specific behaviors of the silica under SFC conditions. Attaching ligands or end‐capping is used to reduce non‐specific interactions on the silica surface; however, many “hot spots” and residual silanol groups may still exist.^6^ Another way to avoid non‐specific interactions is using polymer‐based stationary phases. For instance, Fujito *et al* reported the use of novel highly cross‐linked styrene‐divinylbenzene polymer‐based stationary phase, characterized by hydrophobicity, zero silanol groups, π‐π interaction, high specific surface area, and high durability under SFC conditions for retaining 23 compounds including flavors, aromas, and fragrances.^90^ The stationary phase nature also affects the analyte retention.^13^, ^53^, ^87^, ^91^, ^92^ For examples, the 2‐EP stationary phase is characterized by high polarizability and hydrogen‐bond acceptor interactions, while the poly(vinylpyridine) polymers and the diol stationary phases show especially high hydrogen‐bond donor interactions. The fluorocarbon stationary phases, for instance, the pentafluorophenyl stationary phase (PFP) can be considered alternative reversed phases due to the solvophobicity and fluorophilicity of the fluorinated phases. The advantage of the fluorocarbon stationary phases is the selectivity improvement for the analysis of the organofluorine, polar pharmaceutical compounds, proteins, peptides, nucleotides, steroids, and alkaloids.^93^ The PFP seems to favor the polarizability and dipole‐dipole interactions. For classical well‐endcapped alkylsiloxane bonded stationary phases, increasing the alkyl chain length increases the polarizability and dispersive interactions. At the same time, the increase of alkyl chain length prevents analytes from reaching the stationary phase surface, hence decreasing the dipole‐dipole, hydrogen‐bond donor, and hydrogen‐bond acceptor interactions.^54^, ^94^ The non‐endcapped and polar‐embedded ODS stationary phases can provide the polar interactions due to their large amounts of residual silanol groups on the surface and the attached polar groups, even the well‐endcapped alkylsiloxane stationary phases may exhibit polar interactions. Hence, these stationary phases can provide different selectivities under SFC conditions.^95^ Attaching a phenyl ring to the basis of the ODS chain reduces the bonding density and shields the stationary phase surface better, hence lowering the dispersive interactions. In contrast, attaching the amide group to the ODS chain increases greatly the hydrogen‐bond donor interaction and results in strong interaction with acidic compounds.

Depending on the analytes, pSFC users can consider the relevant structure of the ligand‐bound to the surface of packed material or the relevant molecular interactions provided by stationary phases. For example, the cyano phase shows a strong interaction of accepting hydrogen bonds from analytes; hence, it will not suit for alkanes separation. Both diol and silica phases show donating and accepting hydrogen bond interactions; hence, they can retain well the analytes that have similar interactions. However, if the analytes have different non‐polar structures, they can be better separated on the diol phase due to the strong dispersion interactions of the phase. Recently, the increasing usage of sub‐2 µm fully porous particles or sub‐3 µm superficially porous particles has greatly improved the availability of pSFC stationary phases.^88^, ^92^

### 2.3 pSFC retention behavior

When the modifier percentages in the SFC mobile phase increases, the mobile phase becomes closer to the conventional liquids. Several studies showed that the pSFC retention behaviors in some cases share many similarities with the LC retention behaviors.^53^, ^96^ Other studies also consider pSFC retention mechanisms the combination of the GC and LC retention mechanisms.^86^ Engel *et al* reported that the separation of a mixture of benzene, phenol, *o*‐cresol, *p*‐cresol, *o*‐nitrotoluene, and 3,5‐xylenol using SFC in combination with nonpolar porous glassy carbon packed column.^97^ The authors concluded that on the porous glassy carbon stationary phase SFC showed the same retention mechanism with RPLC, and had more advance for the structural isomers separation. Smith *et al* reported that retentions of alkyl aryl ketones series on the cyano column were the combination of NP interactions and the solute's volatility.^98^ Sakaki *et al* reported that retention of beta‐carotene on the nonpolar stationary phase depended on its solubility in the supercritical mobile phase, and the retention mechanism in the study could be explained by the RPLC theory.^53^, ^96^ In 2019, Hirose *et al* highlighted the differences between the SFC and HPLC retention behaviors of 12 representative compounds including polar compounds with hydroxyl groups, nonpolar compounds, acidic compounds, and basic compounds on several polar and nonpolar stationary phases using scCO_2_ mobile phase for SFC and hexane mobile phase for NPLC.^99^ The results from the study showed that on polar stationary phases (e.g. silica gel, hydroxyphenyl, pyridinyl), pSFC retention behaviors of the polar compounds were very similar to the NPLC retention behaviors involving the hydrophilic and ionic interactions. On low‐polarity phases (e.g. ODS, cholesteryl), both polar and nonpolar compounds were slightly more retained in SFC than in NPLC. The analyte retention behaviors in pSFC showed no statistically significant difference between endcapped and non‐endcapped ODS columns, which indicated the effect came from alkyl chains only, not the residual silanol groups. For the stationary phases that showed strong π‐π and dispersive interactions (e.g. pyrenylethyl, pentabromophenyl), the retention of both polar and nonpolar compounds in pSFC was enhanced several times compared to those observed in NPLC. The authors concluded that the strong dispersive interaction was the reason for the analyte retention on nonpolar stationary phases in pSFC. Regarding the retention shift, the adsorption of the CO_2_, the modifiers, and the additives changes the interactions between analytes and stationary phases. For instance, it was found that increasing the alkyl chain length of the analytes reduced the analyte retentions in cases of using MeOH and acetonitrile (ACN) as modifiers, whereas using isopropanol the effect on analyte retention was inverted.^61^ Tarafder *et al* reported an unexpected retention behavior of octylbenzene, octadecene, anthracene, and pyrene on the silica and ODS stationary phases at the conditions near‐critical region of CO_2_.^86^ The results from the study showed that the retention factors of these compounds at these conditions increased when temperature increased along low‐density isopycnic lines. The authors pointed out the most possible reason was the formation of multilayer adsorption of CO_2_ on the stationary phase surface. Losacco *et al* concluded that depending on the modifier percentage, there were three retention behaviors happened in pSFC when the temperatures change.^84^ Lesellier *et al* reported that when a significant proportion of water is used in the mobile phase, the analyte retention in RPLC was dominated only by the dispersive interaction and the hydrogen‐bond with proton acceptor interaction.^13^ Whereas, despite the stationary phases and the mobile phase compositions, several types of interactions between the analytes and the stationary phases all contributed to the analyte retention in pSFC. Fujito *et al* confirmed that the retention behavior on the NH_2_ stationary phase was strongly affected by the number of hydroxyl groups (OH^−^).^90^ Addition of one OH^−^ on an aromatic acid increases the separation factor on both BEH silica and BEH 2‐EP stationary phases; however, the separation factors on BEH 2‐EP is much better.^95^ Adding one methoxyl group (MeO) next to the OH^−^ group on the aromatic ring, the retention of the compound is decreased on these phases. Surprisingly, adding one more MeO group next to the OH^−^ group again increases the compound retention factor.^100^ For flavonoids, several studies showed that these compounds shared the same retention order on both BEH silica and BEH‐2EP if they shared the same skeleton.^101^, ^102^ Adding one MeO group on their skeleton, the analyte retention is only decreased on BEH‐2EP, while increasing the analyte retention on BEH silica is observed. Lesellier *et al* concluded that on the polar stationary phases increasing the polar groups (eg, OH^−^, acidic, amino groups) increased the analyte retention.^95^ In most cases, the polar compounds retain better on the polar stationary phases, while the hydrophobic ones elute early on these phases.^103^, ^104^ On less polar stationary phases (e.g. cyanopropyl‐ bonded silica), the analyte retention increases with the number of carbon when pure CO_2_ is used.^98^ However, when using significant proportions of modifiers, the analyte retention decreases with the increase of hydrocarbon volume.^61^ On nonpolar well‐endcapped columns, under pSFC conditions, the aromatic and hydrophobic compounds are likely to retain, while the polar compounds do not show much retention. The retention patterns of pSFC somehow are similar to RPLC when a large proportion of organic solvent is presented in the mobile phase.^13^ However, on the mixed‐polarity stationary phases such as non‐endcapped ODS, polar endcapped ODS, polar embedded ODS, and aromatic ligand phases, the pSFC retention patterns are quite distinctive. On these stationary phases, the analyte retention increases when either hydrocarbon molecular volume or solute polarity increases, and the selectivity shows significant changes.^105^, ^106^, ^107^, ^108^ Fujito *et al* reported that the retentions of volatile compounds having multiple double bonds or aromatic rings were strong on the highly cross‐linked styrene‐divinylbenzene polymer‐based column with almost the same retention order obtained on the phenyl column.^90^ Vera *et al* reported the differences in polycyclic aromatic hydrocarbon (PAHs) selectivity on two aromatic columns, that are Synergi Polar‐RP column (ether‐linked phenyl phase with proprietary hydrophilic end‐capping) and Cosmosil 5PBB column (with pentabromobenzyl group bonded silica).^108^ It was found that the dependence of PAHs selectivity and MeOH concentration was only observed on the Synergi‐polar‐RP column, although PAHs retention was stronger on the Cosmosil column. Surprisingly, there is no difference in retention order of flavonoids and estrogens between silica phases and PFP phases. However, for the compounds that have penta rings or hexa rings, the retentions are much better in the PFP phase.^109^, ^110^

While the retention behaviors of achiral compounds are well studied in HPLC, a complete understanding of the pSFC retention mechanism has not been achieved due to complicated effects of the mobile phase density changes, unclear effects of the adsorption happened on the stationary phase, the complex combination of several interaction types, and the imperfections of the instrument.^6^ Another problem is that in the past many studies were conducted using preparative‐scale instruments. To overcome these problems, many efforts have been made by using the new analytical‐scale instruments or combining several modeling approaches to study the retention behaviors.

## 3 pSFC RETENTION MECHANISM STUDIES

Different studies have been conducted to give a better understanding of the retention mechanisms in pSFC, for example, explaining the processes inside the column under SFC conditions, or establishing the relationships between the analytes, the mobile phases, the stationary phases, and the retention behaviors.^6^ These studies can provide more knowledge for the use of supercritical fluids in practical separation science, for instance, contributing better decisions on the choice of suitable columns and chromatographic conditions that fit analysis purposes.^99^, ^111^ Moreover, a good predicting model can reduce the wrong annotation regarding the nontargeted analysis or help the evaluation of the properties of unknown compounds such as the lipophilicity, dissociation constants, and relative bioactivities.

Many conclusions regarding pSFC retention mechanism were made based on the establishment of good and statistically significant models. The important statistical information of a model are the goodness‐of‐fit and predictive ability.^112^ In which, the goodness‐of‐fit expressed in the term of *R* ^2^ (the coefficient of determination), indicates how well the model fits a specific dataset. A value *R* ^2^ of 0.80 simply means that it is possible to explain 80% of data variation with the proposed model. The *R* ^2^ value falls in the range of 0‐1, where 0 means no fit at all and 1 means a perfectly fitting model. For prediction purposes, the predictive ability of a model is even more important. The predictive ability expressed in the term of *Q* ^2^ (the goodness of prediction) indicates how good the prediction is internally via an existing dataset (cross‐validation) or externally by using another independent validation set.^113^ The value of Q^2^ also is in the range of 0‐1, where a value of more than 0.5 can be considered as good, and the value of more than 0.9 is excellent. As a rule of thumb, a high *R* ^2^ value is needed to achieve a high *Q* ^2^ value; however, the difference between *R* ^2^ and *Q* ^2^ should not exceed 0.2‐0.3. Some common cross‐validation strategies for determining *Q* ^2^ are leave‐one‐out, holdback, and K‐fold. The use of each strategy depends on dataset characteristics, for example, the leave‐one‐out may not be recommended for a dataset that has many samples.^113^ Regarding pSFC retention mechanisms studies, despite the purposes, complexities, and requirements, most of the studies can be classified into three main categories, which are empirical approaches (EM), thermodynamic approaches (TM), and extra thermodynamic approaches (ETM).^10^, ^114^, ^115^

### 3.1 Empirical approaches

Regarding the SFC retention mechanism, Yonker stated, “Retention in supercritical fluid chromatography is a complex function of temperature, pressure, and solute concentration”.^62^ Many studies applied EM that predicts the behaviors of a system based on empirical observations to explain pSFC chromatographic behaviors of many compound groups (e.g. tocopherols, alkanes, PAHs, hexadecane, fluoranthene, phenolic derivatives, carboxylic methyl esters, triglycerides, organic acids, amines, and amides).^114^, ^116^, ^117^, ^118^, ^119^, ^120^, ^121^, ^122^, ^123^, ^124^, ^125^, ^126^, ^127^, ^128^

Mitra *et al* reported the analyte retention in SFC was a function of the reduced density, which is the ratio of the actual mobile phase density to its density at the critical point, and the reduced temperature, which is the ratio of the actual mobile phase temperature to its critical temperature.^114^ In the study, the analyte retention factors were described by the following equations:

(1) $$\ln k^{\prime }=\;a+b\rho_{R}+cT_{R}$$

(2) $$\ln k^{\prime }=\;a+b\ln\rho_{R}+cT_{R}$$

where *a, b*, and *c* are the constants, *ρ_R_* is the mobile phase reduced density, and *T_R_* is the mobile phase reduced temperature. Using the same dataset, in comparisons with two candidate models proposed by Martire and Sakaki, the model proposed by Mitra was less complex and had higher R^2^ values with lower standard errors.^53^, ^129^ In 2018, Enmark *et al*. applied Design of Experiments (DoE) strategy to investigate the robustness of gramicidin separations on a hybrid silica column (ie, Kromasil SFC‐ 2.5‐XT) using a mobile phase consisted of CO_2_, water, and MeOH under isocratic and gradient modes.^130^ The authors successfully assessed the method robustness, and revealed the relationship between the retention and four factors affecting the analyte retention. In the study, the analyte retention factors in isocratic mode was described by the following equation:

(3) $$\log_{10}\left(k\right)\;=\;\alpha_{1}\;P\;+\;\alpha_{2}C_{tot}\;+\;\alpha_{3}T\;+\;\alpha_{4}C_{H_{2}O}\;+\;\alpha_{5}T^{2}\;+\;\alpha_{6}PT\;+\;\alpha_{7}TC_{H_{2}O}\;+\;\beta$$

where *C_tot_* is the co‐solvent fraction; *C_H2O_* is the water mass fraction in the co‐solvent; *P* is the pressure, and *T* is the column temperature. In cases of constant pressure and temperature, equation 3 was simplified as:

(4) $$\log_{10}\left(k\right)=\alpha_{2}\;C_{tot}+\left(\alpha_{4}+\alpha_{7}T\right)C_{H_{2}O}+\gamma$$

where *k* is the retention factor; *α_2_, α_4_, α_7_* are the coefficients; *β and γ* are the constants. When a linear gradient was applied, the analyte retention time was described by equation 5:

(5) $$t_{R}=\frac{t_{0}}{G}\;\left(Gk_{start}+1\right)+t_{0}$$

(6) $$G\;=\;S\Delta \phi \frac{t_{0}}{t_{g}}$$

where *t_g_* is the gradient time; *t_0_* is column void time; *G* is gradient steepness factor; *k_start_* is the retention factor at the beginning of gradient, and *∆ϕ* is the change in total co‐solvent during the gradient. It was found that in both isocratic (eq. 4) and gradient (eq. 5) modes, the *C_tot_* and *C_H2O_* were the two most important factors that contribute to the analyte retention. Moreover, under a high mass fraction of MeOH and H_2_O as co‐solvents, a small density variation over a large range of pressure and temperature could be obtained, hence boosting the method robustness.^130^ Furthermore, when employing the gradient elution, increasing the gradient slope also increased the separation robustness.

### 3.2 Thermodynamic approaches

Although EM helps to predict the chromatographic behaviors of certain compound groups under specific analyzing conditions, the TM are proved to provide a better insight into the theoretical background of pSFC retention mechanisms.^10^ The magnitude of thermodynamic parameters used in the TM usually expresses the combination of possible individual interactions at the molecular or sub‐molecular level, hence reflecting the net interactive effects of a given system.^131^ Several thermodynamic parameters used in the TM for the retention behavior modeling are the diffusion coefficient, partial molar volume, compound adsorption properties, enthalpy and entropy of transfer of the solutes from the mobile phase to the stationary phase, and so forth.^53^, ^132^

In 1990, Grover *et al* used TM to predict pSFC retention time of analytes with errors of less than 20% based on the well‐depth of the surface site interaction potential, $u_{b\;}^{0},\;$on ODS and Alox‐T columns employing pure CO_2_ as mobile phase.^115^ Four years later, Sakaki *et al* applied a free‐energy‐change of solute transfer equation to study pSFC retention behavior of β‐carotene on both polar and nonpolar columns (ie, Inertsil ODS, Cosmosil C_18_ AR, W2‐100P, Cosmosil NH_2_, and Super NH_2_) under isocratic mode at various temperatures and pressures.^53^ The authors concluded that the analyte retention on the nonpolar columns was a function of its solubility in the mobile phase described by equation 7, while the analyte retention factor on the polar columns could be described by the equation proposed by Bartle *et al* (equation 8).^53^, ^133^, ^134^

‐For nonpolar stationary phases:
(7) $$\ln k^{\prime }=\ln\phi \;+\ln C_{st}^{o}-C-k\ln\rho +\frac{\mu_{ss}^{o}-\mu_{st}^{o}}{RT}+\frac{\Delta H_{m}^{o}}{RT}$$
‐For polar stationary phases:
(8) $$\ln k^{\prime }=\ln\phi \;+\ln C_{st}^{o}+\frac{\left(\mu_{ss}^{o}-\mu_{st}^{o}\right)}{RT}-\ln S$$

where *k’* is the capacity factor; lnϕ is the volume ratio of the stationary phase and mobile phase; $C_{st}^{o}$ is the standard (surface or volume) concentration in the stationary phase; *C* is a constant; *k* is the number of solvent molecules, associating with one solute molecule; *ρ* is the gas density (kg/m^3^); $\mu_{ss}^{o}$ is the chemical potential of the pure solid solute at standard pressure; $\mu_{st}^{o}$is the chemical potential of the solute in the stationary phase as it moves toward infinite dilution and standard concentration and pressure; $\mu_{ss}^{o}-\mu_{st}^{o}\;$is the free‐energy‐change, relating to the solute transfer from the solid‐state into the stationary phase; ∆*H°_m_* is the value of total reaction enthalpy (solvation and vaporization); *Τ* is the temperature (K); *R* is the gas constant (8.314 J.mol^−1^.K), and *S* is the solubility of β‐carotene (g/m^3^). The authors noted that β‐carotene retention was less affected by residual silanol groups on the silica surface due to the nonpolarity and bigger size (compared to C_18_‐ groups) of the analyte. It also means that the effect of the compound shape was ignored; hence, the equations may not work well in other cases. Roth *et al* first described the pressure dependence of the compound retention in a hypothetical, isothermal, isobaric SFC system.^135^ Later, the authors proposed another thermodynamic equation to explain the dependence of the retention change and the change in the composition of a binary mobile‐phase.^136^ In the study, the retention change was described by the following equation:

(9) $$\Delta \ln k_{1}\approx \Delta \ln\varphi_{1m}^{\infty }-\xi_{4m}\Delta_{x_{4m}}-\frac{V_{s}}{V_{m}}\xi_{4s}\Delta_{x_{4s}}-\frac{1}{RT}{\left(\frac{\partial \mu_{1s}^{\infty }}{\partial_{x_{4s}}}\right)}_{T,P,n_{2s}}\Delta_{x_{4s}}$$

where *k_1_* is the retention factor; ∆x_4s_ is the change in modifier fraction in the stationary phase causing by changes in compositions of the mobile phase fluid; $\varphi_{1m}^{\infty }$ is the infinite‐dilution fugacity coefficients of the compound in the two phases; *V_s_* is the geometric volume of the stationary phase, and ζ_4m_ mixing expansivity. The equation 9 highlighted the great impact of mobile phase composition changes on retention factor even when interfacial adsorption could be ignored. Moreover, the authors concluded that the analyte retention was driven only by bulk partitioning.

In 2011, Kaczmarski *et al* proposed the numerical modeling of the elution peak profiles for a series of alkane (from C1 to C18) on 5 µm Waters Spherisorb‐C8 particles under SFC condition.^72^ The proposed model consists of three sub‐models, for example, the heat transfer model, mass transfer model, and mobile phase velocity distribution model. Among the sub‐models, the mass transfer model was the most important. This sub‐model included all equations accounting for the temperature, viscosity, velocity, density, external porosity, and packing density gradients, hence explaining the propagation of the alkane band along a column. However, the proposed model required the estimation of the external heat transfer coefficient, which could be done by measuring the column wall temperature at the highest mobile phase flow rate.

‐The general mass balance model was described in the following equation:
(10) $$\frac{\partial C_{A}}{\partial t}+F\frac{\partial q_{A}}{\partial_{t}}+\frac{1}{\varepsilon_{t}}\;\frac{\partial (uC_{A})}{\partial z}=\;-\frac{\partial }{\partial z}\left(Jz\right)-\frac{1}{r}\frac{\partial }{\partial r}\left(rJ_{r}\right)$$

where *C_A_* is the analyte concentrations in the MP; *q_A_* is analyte concentrations in stationary phase at equilibrium (g/L); *t* is the time; *z* is the axial coordinate; *u* is the velocity of axial superficial MP; *ε_t_* the total porosity of the column, and *F* is the phase ratio. The *J_z_* and *J_r_* account for axial and the radial mass flux components, which were described by the following equations:

(11) $$J_{z}=\;-D_{z,a}\frac{\partial C_{A}}{\partial_{z}}$$

(12) $$J_{r}=\;-D_{r,a}\frac{\partial C_{A}}{\partial_{r}}$$

where *D_z,a_* and *D_r,a_* are local axial and radial apparent dispersion coefficients (m^2^/s) respectively, explained by the following equations:

(13) $$D_{z,a}=\frac{D_{L}\varepsilon_{e}}{\varepsilon_{t}}\;+{\left(\frac{k_{1}}{1+k_{1}}\right)}^{2}+\frac{u^{2}d_{p}}{\varepsilon_{t}\varepsilon_{e}F_{e}6}\left[\frac{d_{p}}{10D_{eff}}+\frac{1}{k_{ext}}\right]$$

(14) $$D_{r,a}=\frac{0.03d_{p}u}{\varepsilon_{t}}\;+0.7d_{m}$$

(15) $$k_{1}=F_{e}\;\left(\varepsilon_{p}+\left(1-\varepsilon_{p}\right)\frac{\delta q_{A}}{\delta C_{A}}\right)$$

(16) $$F_{e}=\frac{1-\varepsilon_{e}}{\varepsilon_{e}}\;$$

(17) $$D_{eff}=\frac{D_{m}\varepsilon_{p}}{\tau }\;$$

where *D_L_* is the axial dispersion coefficient; *d_p_* is the adsorbent diameter; *D_eff_* is effective particle diffusivity; *ε_e_* is the external porosity; *ε_p_* is the particle porosity; *k_ex_*_t_ is the external mass transfer coefficient, and *τ* is the tortuosity coefficient calculated by the following equation:

(18) $$\tau \;=\frac{{\left(2-\varepsilon_{p}\right)}^{2}}{\varepsilon_{p}}$$

‐The local value of mobile phase velocity was calculated by the following equation:
(19) $$U_{z}\;\left(r,z\right)=\frac{u^{o}\rho^{o}}{\eta \left(r,z\right){\left(\bar{p/\eta }\right)}_{z}}\;\frac{\varepsilon_{e}{\left(r,z\right)}^{3}}{{\left(1-\varepsilon_{e}\left(r,z\right)\right)}^{2}}$$

where *u^o^* is the mobile phase superficial velocity; ρ *^o^* is the mobile phase density at the column inlet; *η* is the viscosity, and ($\bar{\rho /\eta }$) is the average value at a given axial position, calculated by the following equation:

(20) $${\left(\frac{\bar{\rho }}{\eta }\right)}_{z}=\frac{2}{R_{i}^{2}}\;\int_{0}^{R}\frac{\rho \left(r,z\right)}{\eta \left(r,z\right)}\frac{\varepsilon_{e}{\left(r,z\right)}^{3}}{{\left(1-\varepsilon_{e}\left(r,z\right)\right)}^{2}}r\;dr$$

‐Later, the external column porosity was assumed to reduce along the column according to equation 21:
(21) $$\varepsilon_{e}=\;A{\left(\frac{z}{L}\right)}^{N}+B$$

The proposed numerical model combined heat balance equation and mass balance equation resulted in good agreement with the measured column wall temperature and the pressure at the column outlet. It was confirmed that the proposed model enabled excellent prediction of temperature and pressure profiles inside the column, retention time, and band profiles of un‐sorbed‐ and sorbed‐ alkanes at highly reduced density and high flow rate. Moreover, the authors concluded that at low reduced densities, the column axial distribution of porosity showed a high impact on the band profiles. However, the proposed model could not predict accurately the retention of the sorbed analytes at low reduced densities and fast flow rates, which might be caused by the impact of the axial distribution of the external porosity.^72^ Later work from Leśko *et al*. applied an inverse method for estimating the linear isotherm model parameters based on retention time of n‐octadecylbenzene on a Luna C18 column operated under convective and still air conditions employing 95% CO_2_/5% methanol (v/v) as mobile phase.^137^ To account for the radial and axial distribution of physicochemical parameters along the column at condition near the critical pressure, the two‐dimensional heat balance and mass balance models were considered. The authors concluded that the presence of a significant‐high outlet pressure allowed ignoring the radial gradients; therefore, the application of the one‐dimensional models was adequate. In this study, the proposed one‐dimensional models included the following sub‐models:

‐The one‐dimensional heat balance model, described by the following relationship:
(22) $$c_{p}^{m}u_{z}\;\frac{\partial T}{\partial z}=\frac{\partial }{\partial z}\;\left(\lambda_{ef}\frac{\partial T}{\partial z}\right)-u_{z}\left(1-\alpha T\right)\frac{\partial P}{\partial z}+h\left(T_{ext}-T\right)$$

where $c_{p}^{m}$ is the mobile phase heat capacity (J/m^3^/K); *T* is the average mobile phase temperature inside the column at the position *z* (K); *λ_ef_* is the effective bed conductivity (W/m/K); *α* is coefficient of thermal expansion (1/K); *P* is pressure (bar); *h* is the effective heat‐transfer coefficient (W/m^3^/K), and *u_z_* is the mobile phase superficial velocity in the axial direction (m/s).

‐The one‐dimensional mass balance model was described by the following equation:
(23) $$\frac{\partial c}{\partial t}+F\frac{\partial q}{\partial t}+\frac{1}{\varepsilon_{t}}\;\frac{\partial \left(u_{z}c\right)}{\partial z}=\frac{\partial }{\partial z}\;\left(D_{z,a}\frac{\partial c}{\partial z}\right)$$

where *c* and *q* are molecule concentrations in the mobile phase and the stationary phase (g/L); F = (1 − ε_t_)/ε_t_ is phase ratio; *ε_t_* is total column porosity, and *D_z,a_* is local apparent dispersion coefficient in the axial direction (m^2^/s).

‐The mobile phase velocity and pressure distribution model was calculated from the following equation:
(24) $$-\;\frac{\delta P}{\delta z}=\;\xi \frac{u^{o}\rho^{o}{\left(1-\varepsilon_{e}\right)}^{2}\eta }{\varepsilon_{e}^{3}d_{p}^{2}\rho }$$

where *u^o^* and ρ ^o^ are superficial velocity and density of mobile phase, respectively, calculated at column inlet; ξ is the empirical parameter with the value of 150; *d_p_* is particle diameter; *ε_e_* is external porosity, and the axial velocity is described as:

(25) $$u_{z}\left(z\right)\;=\frac{u^{o}\rho^{o}}{\rho \left(z\right)}\;$$

‐The linear isothermal model:
(26) q=Hc

where *H* is the Henry constant described by the following equation:

(27) $$H\;=\;exp\left(c_{0}+\frac{c_{1}}{T_{r}}+c_{2}\rho_{r}+c_{3}\frac{\rho_{r}}{T_{r}}+c_{4}\frac{\rho_{r}^{2}}{T_{r}}\right)$$

where *T*_r_ and ρ_r_ are the mobile phase reduced temperature and mobile phase reduced density, respectively; *c_0_ – c_4_* are the coefficients, calculated from experimental data. The one‐dimensional models showed a good agreement between theoretical and experimental retention time with errors of less than 3% in 93% of experiments under still air condition. Moreover, the sub‐models could be applied successfully for accurate estimation of the pressure, temperature, and velocity distribution under this condition.^137^ Nevertheless, the study highlighted the difficulties in modeling the radial temperature gradients under convective air mode.

In 2015, Tyteca *et al* investigated the use of non‐linear mixed‐mode HILIC and RP retention models in SFC. The authors proved the non‐linear dependence of SFC retention factor on the fraction of the organic solvents and the natural logarithm of the fraction under both isocratic and gradient modes.^138^, ^139^, ^140^ In the study, the analyte retention factor could be described by the following models:

‐The nonlinear mixed‐mode HILIC retention model was described by the following equation:
(28) $$\ln\;\left(k\right)=\;\ln\left(k_{w}\right)+S_{1}\phi +S_{2}\ln\left(\phi \right)$$
‐The nonlinear RP retention models, were described by the following equations:
(29) $$\ln\left(k\right)=\ln\left(k_{w}\right)\;+S_{1}\varphi +S_{2}\phi^{2}$$
(30) $$\ln\left(k\right)=\ln\left(k_{w}\right)\;+2\ln\left(1+S_{2}\phi \right)-\frac{S_{1}\phi }{1+S_{2}\phi }$$

where *ϕ* is the fraction of water, *k_w_* the extrapolated value of *k* for *ϕ* = 0 (eg, pure CO_2_), *S_1_* is the slope, and *S_2_* is the curvature coefficient. Four columns with the same dimension and particle size from Waters were used in this study, including Acquity UPC^2^ BEH (BEH), Acquity UPC^2^ BEH 2‐EP (2‐EP), Acquity UPC^2^ HSS C18 SB (HSS), and Acquity UPC^2^ CSH Fluoro‐Phenyl (PFP). The mobile phase B was 98% MeOH/2% water (v/v) with 10 mM ammonium formate. First, the nine tested compounds including acidic, basic, and neutral compounds were used to test the models under isocratic mode on the BEH, 2‐EP, and HSS stationary phases. The models showed high values of goodness‐of‐fit ($R_{adjusted}^{2}$) but lower goodness‐of‐prediction (*Q^2^*) due to prediction difficulty of asymptotic behavior on the BEH stationary phase. However, in cases of more than 20% of B was used, the model could not predict accurately retention time for the least retained compounds. Despite this disadvantage, the authors concluded that the isocratic SFC retention behavior could be accurately expressed by both nonlinear mixed‐mode HILIC (eq. 28) and RP (eq. 29) retention models.^140^ Later, all models were used to predict retention for the same compound set under several gradient settings, which resulted in a quite good prediction with the prediction errors in the range of ±10%. Lastly, individual retention time modelling based on the equation 30 were used to optimize the gradient of a 16 components mixture including eight drugs and their main phase I metabolites. The best linear gradient was successfully obtained, while still giving accurate prediction of retention time with errors of less than 5% under optimum condition.

Gritti *et al* proposed a simplified, practical heat‐transfer model to estimate and predict the radial temperature profiles across a column packed with fully porous HSS SB C18 particles using a low‐density CO_2_ mobile phase.^141^ The proposed model helped to maximize column efficiency for analyzing several semi‐volatile compounds when employing low‐density mobile phases. In the model, equation 31 was used to estimate overall amount of viscous and expansion heat dissipated per unit time along the column:

(31) $$P_{heat}=\left(1+〈\alpha T〉\right)\;F_{v}\Delta P=\left(1+〈\alpha T〉\right)\;\frac{180{\left(1-\in_{e}\right)}^{2}}{\in_{e}^{3}d_{p}^{2}}\frac{\eta L}{\pi r_{c}^{2}}{F_{c}}^{2}$$

where 〈$F_{v}$〉, 〈η〉, and 〈αT〉, are the average flow rate, viscosity, product of the temperature by the thermal expansion coefficient of mobile phase; *∈_e_* is the interstitial porosity of the packed bed, and *d_p_* = 1.8 µm is the average particle diameter. The simplified equations of the temperature difference between the outlet and inlet of chromatographic bed (*∆_L_T*), and temperature difference between the center and the wall of the chromatographic bed *(∆_R_T*) were described by the following equations:

(32) $$\Delta_{L}T\;=\;\left(1+f\right)\left(1+\langle \alpha T\rangle \right)\frac{\Delta P}{\left\langle C_{p}\right\rangle }$$

(33) $$\Delta_{R}T\;=\;-f\left(1+\langle \alpha T\rangle \right)\frac{\langle F_{v}\rangle \Delta P}{4\pi L\langle \lambda_{eff}\rangle }$$

where 〈$\lambda_{eff}$〉 is the average effective thermal‐conductivity of the packed bed when it is immersed in the SFC mobile phase. Finally, the radial heat‐flux crossing any concentric cylinder of length L under SFC conditions was described by the relationship:

(34) $$\Phi =-f\left(1+\langle \alpha T\rangle \right)\left\langle F_{v}\right\rangle \Delta P>0$$

The proposed heat transfer model was able to predict intensive steepness of the temperature gradients across the column diameter. The authors concluded that the effect of the radial density gradient across the column diameter could be compensated by properly adjusting the difference between the mobile phase temperature and the column temperature.^141^ Moreover, the maximum column efficiency could be achieved by complete thermal insulation of the column under a high vacuum.

Using a simple model based on a virtual fluid, Poe *et al* showed that under studied conditions Joule‐Thomson coefficient, which related to the temperature change of a gas or liquid forcing through the porous medium while there was no heat exchange with the environment, played an important role in efficiency losses of n‐alkylbenzenes elution on both fully porous and superficially porous particles packed columns.^40^ Moreover, the effects of mobile phase penetrating through a porous medium under pressure gradient causing viscous heating and enthalpic expansion were also included in the model. Nevertheless, the authors concluded that the virtual fluid models only explained the column efficiency losses caused by radial temperature gradients and provided no information regarding the shape of band profiles.

### 3.3 Extrathermodynamic approaches

The TM are important and provide useful information regarding the theoretical background of pSFC retention mechanisms. However, the TM are usually complex and require specific parameters and constants which are not easily obtained.^114^ Moreover, the TM are usually studied under “ideal” conditions that are quite different from practical experiments, that is, using simple mobile phase compositions, isocratic mode, a small dataset. Additionally, TM usually limits in providing physical chemistry information and in general rarely applied in practical separation science.^10^ Hence, ETM is further used to establish the empirical correlation between thermodynamic parameters with the molecular structures, chromatographic conditions, and enthalpy‐entropy compensation.^10^, ^142^ Some ETM based on the quantitative structure–retention relationships (QSRR), which employ the molecular descriptors that well reflect physicochemical properties of a representative compound group to establish the relationship between compound properties and chromatographic retention data of these compounds.^61^ To obtain reliable QSRR models, choosing suitable parameters is important, and a strict statistical analysis must be proceeded.^131^ The assumption that all similar molecules should have similar activities is strongly applied here. Thus, the main problem is how to describe the minor differences between the molecules, since the differences may cause variation in the analyte retention. Several molecular descriptors have been proposed and used in QSRR such as the topological indices (real numbers that represent aspects of molecular structure), the autocorrelation descriptors (unique for a given molecular geometry, independent to translation and rotation), the electrotopological‐state indices (including both electronic and topological features), and the linear solvation energy relationships (LSER) descriptors.^112^, ^143^, ^144^, ^145^, ^146^ More than 1500 molecular descriptors of a specific molecule can be easily obtained by many software (e.g. PaDEL‐Descriptor, DRAGON, BlueDesc). There is no clear rule that defines which parameters should be included in the models. Ideally, all reliable quantitative parameters can be subjected for the construction of the relationship and to get the statistically significant relationships.^147^ However, the highly correlated descriptors, which do not contribute to retention explanation and only add noise to the model, must be avoided.^148^ Among QSRR models, LSER models recently have become dominant in the study of the retention mechanism of pSFC.

#### 3.3.1 Linear solvation energy relationships

The LSER models proved it as a powerful tool to characterize the interactions that happened in pSFC.^149^, ^150^, ^151^ Briefly, LSER describes the linear relationship between the logarithm of retention factor and several fundamental intermolecular interactions that occurred in the system. The basic LSER model using Abraham solvation parameters can be described by the following equation:

(35) logk=c+sS+aA+bB+vV

where *k* is the solute retention factor; *E, S, A, B*, and *V* are the solute descriptors; *e, s, a, b*, and *v* are the system constants providing information about the relative selectivity of the system toward a specific molecular interaction; *c* is the solute‐independent constant, specific for the column; *E* is excess molar refraction representing for polarizability contributions of *n* and *π* electrons; *S* is the dipolarity/polarizability of a solute; *A* and *B* are the overall hydrogen‐bond acidity and basicity of solute; *V* is the McGowan characteristic volume of solute (cm^3^.mol^−1^/100) representing both the endoergic cavity formation process and the exoergic dispersion interactions.^151^ Several compounds with known solute descriptors were measured using multiple linear regression analysis to obtain the constants of a certain system. If a system coefficient is positive, the corresponding specific interaction with the stationary phase is more pronounced, hence explaining the increase of solute retention time. If the negative value of the system coefficient is obtained, the specific interaction is more favorable by the mobile phase, which in turn reduces the retention. Regarding the use of LSER to study pSFC retention mechanism, in 1997, Blackwell *et al* conducted a series of experiments regarding the effects of different parameters on pSFC retention of 1‐substituted naphthalene derivatives.^152^ The results successfully predicted retention time of some tested compounds at conditions near the mobile phase critical condition and showed the potential of controlling the mobile phase eluotropic strength and the column efficiency. Fu *et al* reported that the relationship between the bonding density, the stationary phase dispersion interaction, and polar interaction when analyzing five synthesized non‐endcapped C8 stationary phases that had different bonding density and carbon content.^153^ The authors also concluded that adding modifiers (e.g. MeOH, EtOH, isopropanol, ACN) only affected hydrogen bonding interaction by changing the *a* and *b* coefficients. Pyo *et al* applied LSER to explain the retention behaviors on an ODS‐2 stationary phase of 35 compounds including benzene derivatives, and some organic acids under isocratic mode with the modifier of MeOH.^150^ The obtained models for various modifier percentages (0‐9%) showed good correlation coefficients ranging from 0.0957 to 0.9987 with low standard deviations ranging from 0.0157 to 0.0230. Furthermore, the results from the study revealed that the value of *b* and *s* coefficients greatly decreased when increasing MeOH modifier from 0% to 2%. When more than 2% of MeOH was used, only a gradual decrease of *b* and *s* could be observed. This indicated that at the low MeOH percentages, the modifier coated the free silanols on the surface of the ODS stationary phase, hence changing the retention at these conditions. West *et al* achieved a decent retention model for 51 derivatives of benzene and naphthalene on the porous graphitic carbon column under several isocratic conditions.^151^ Although the model did not explain the retention behaviors on porous graphitic carbon phase as well as it did for ODS stationary phases, it was found that the retention was contributed mainly by polarizability (*E*) and the volume (*V*) of solute. In 2008, Bui *et al* attempted to investigate pSFC retention behavior for more than 200 pharmaceutical and drug‐like compounds employing CO_2_–MeOH as the mobile phase under several isocratic conditions on the cyanopropyl, propanediol, 2‐EP, and amino stationary phases.^154^ The proposed LSER model well fitted to the dataset with high *R^2^* value, revealed that the hydrogen bond acidity and basicity interactions contributed most significantly to the analyte retention. Later, West *et al* further investigated the effects of the mobile phase compositions on the retention and selectivity of more than 100 compounds using several stationary phases (e.g. cyanopropyl‐, pentafluorophenyl‐propyl‐, phenyl‐hexyl‐, phenyl‐ oxypropyl‐, phenyl‐propyl‐, pyridine‐ethyl‐, and ODS‐bonded silica).^61^ The obtained models showed a good correlation between the retention and the analyte LSER coefficient with maximum adjusted determination coefficients ($R_{adj}^{2}$) of 0.995 in case of the ODS column and MeOH as modifier. However, the prediction powers of the models were not assessed. Galea *et al* once pointed out that many LSER studies have been done only with neutral or nearly neutral compounds due to the model lacks terms for ionic interactions.^155^ Hence, West *et al* applied a modified version of the LSER with two additional descriptors D^−^ (the negative charge of anionic and zwitterionic species) and D^+^ (the positive charge of cationic and zwitterionic species) to investigate SFC retention characteristics of PFP stationary phase.^156^ The modified LSER model can be expressed by the following equation:

(36) $$\log k=\;c+sS+aA+bB+vV+d^{-}D^{-}+d^{+}D^{+}$$

where D^−^ and D^+^ account for anionic and cationic interactions, described by the following equations:

(37) $$D^{-}=\frac{10^{\left(pH*-pK*\right)}}{1+10^{\left(pH*-pK*\right)}}\;$$

(38) $$D^{+}=\frac{10^{\left(pK*-pH*\right)}}{1+10^{\left(pK*-pH*\right)}}\;$$

Moreover, the three‐dimensional structure of the analytes can affect their way of interaction with the stationary phase.^53^, ^157^, ^158^ It was found that the “bulky” molecules in comparison to “rod‐like” molecules might have more difficulty penetrating narrow slots in a stationary phase that had “crowded” ligands. Recently, West *et al* used an extended model of LSER with *F* and *G* coefficients (eq. 39) that account for flexibility and sphericity of analyte molecules, to characterize two famous enantioselective stationary phases based on retention behavior of more than 200 achiral compounds using pSFC under isocratic mode with 90% CO_2_/10% MeOH (v/v) as mobile phase.^159^

(39) $$\log k=\;c+sS+aA+bB+vV+d^{-}D^{-}+d^{+}D^{+}+fF+gG$$

where *F* and *G* represent the molecule flexibility and the molecule sphericity, respectively; *f* and *g* are system constants that correspond for the above interactions. The up‐to‐date LSER models (eq. 39) showed only acceptable fit to the dataset used in the study with a maximum value of adjusted determination coefficient ($R_{adj}^{2}$) of 0.856 and a maximum standard error value of 0.280. Nevertheless, regarding the analyte retention the authors concluded that for the cationic species the dipole‐dipole, hydrogen bonding, and ionic interactions were less important compared to the π–π interaction.^159^ Furthermore, the results indicated that small spherical molecules tended to retain stronger than expected because they were more preferable by the chiral selector cleft. Andri *et al* combined the DoE of operating‐conditions and LSER coefficients to establish a global SFC retention time prediction model for 15 pharmaceutical compounds under gradient conditions.^160^ The study applied the partial least square technique successfully proposed well‐fit and robust linear retention models that included the following parameters: the logarithm (base 10) of the partition coefficient (log*P*), experimental factors (i.e. the gradient slope, backpressure, and column temperature), and the LSER coefficients. The results from the study showed a highly agreement between the predicted and the experimental data with an *R^2^* value of 0.942 and a slope value of 1.004 for the tested compounds. Moreover, the proposed global model highlighted the impact of compound physicochemical properties on the retention behavior under gradient conditions. In 2020, Jiang *et al* studied the retention mechanism for a series of synthesized phenyl‐type stationary phases with different substituted benzenes using original LSER models with a set of more than 90 compounds.^161^ The obtained LSER models showed a good fit to the data set with a maximum correlation coefficient value of 0.992 and a maximum standard deviation of 0.255. In addition, it was found that compared to the ODS, the phenyl‐type stationary phases were able to provide all types of interaction with different strengths, and thus can provide a wide range of selectivity. Lastly, by adjusting substituents on benzene, the hydrogen bonding and dipole‐dipole interactions of the stationary phase could be adjusted to widen the application in SFC.

Regarding retention mechanism studies, LSER models proved the power to give a deeper insight in terms of molecular interactions between compounds and pSFC systems. However, most of the available models still focus on explanation the retention behaviors for a set of compounds under isocratic mode due to the LSER coefficients are easier to obtain under these conditions; hence, the effects of the mobile phase gradient on the retention behaviors are ignored. By combining the DoE of the operating conditions and the LSER coefficients, the combined effects of these factors on the analyte retention could be better assessed. Although LSER coefficients can describe the interactions that molecules provide within specific chromatographic systems, the coefficients are difficult to obtain and do not contain much information regarding analyte structure. Moreover, the LSER model was not successful to explain the retention behaviors in some stationary phases, which indicated imperfection of the approach. The LSER coefficients might not be capable to describe interactions in new stationary phases, or the choice of the tested compounds was simply not good enough. Finally, there has been no full guidance on how to obtain LSER coefficients, to construct, and to validate the LSER model.

#### 3.3.2 Other strategies

In 1994, Heaton *et al* reported a universal retention prediction model for a series of aromatic compounds using the multicomponent solubility parameters.^162^ These solubility parameters described the ability of a solute involving the dispersive, dipole‐dipole, dipole‐induced dipole, acidic hydrogen‐bonding, and basic hydrogen‐bonding interactions. The retention of the aromatic compounds was obtained on several packed columns, for example, C_18_‐, phenyl‐, nitrile‐, amino‐bonded phases, and bare silica phase, and could be described by the following equation:

(40) $$\ln k^{\prime }=V_{m}\left(a\delta_{d}+b\delta_{o}+c\delta_{in}+d\delta_{a}+e\delta_{b}+f\right)$$

where *V_m_*, is the molar volume; *k'* is the capacity factor; $\partial_{d}$,$\;\partial_{o},\partial_{in},\partial_{a},\;\mathrm{and}\;\partial_{b}$ are dispersive, dipole‐dipole, dipole‐induced dipole, hydrogen‐bonding acidity, and hydrogen‐bonding basicity parameters; *a, b, c, d, e*, and *f* are weighting coefficients corresponding for respectively solubility parameters of a specific stationary and mobile phase at a given temperature. A multiple regression analysis of a molecule series with general formulas of Ph‐X and C_6_H_4_XY was conducted under several percentages of modifiers (0‐20%) to calculate the weighting coefficients. It was found that the changes in MeOH percentages in the mobile phase caused the variation of coefficient values.^162^ Moreover, when the MeOH percentages changed from 0% to 1%, the coefficients varied significantly. However, when more than 1% of MeOH was used, only a gradual variation of the coefficient values was observed. This observation confirmed the formation of a thin layer of MeOH on the stationary phases. The proposed model successfully predicted the retention order for ortho‐, meta‐, and para‐ of several di‐substituted benzenes on the C18 column. However, the problems of the model were the fluctuations in calculated coefficients and the overestimation in some cases, which were caused by the model inadequacies or the small dataset. Nevertheless, the capability of the model to apply for any type of analytes was very promising.

Tarafder *et al* applied an isopycnic plot together with a previous model to describe pressure and density drops along column length with different mixtures of CO_2_ and MeOH.^57^ In the study, the pressure drop and density drop along the column were described as the functions of outlet pressure and column temperature (eq. 41):

(41) $$\frac{dP}{dz}=\;-\frac{\eta }{\rho }\frac{G}{KS}$$

where *G* is the mass flow rate along the column under steady‐state conditions (assumed to be constant); *S* is the column cross‐section area (assumed to be constant); *K* is the bed permeability; *η* and *ρ* are the viscosity and local density of the mobile phase. The critical temperatures and critical pressures of the mixture were obtained from the following equations (eq. 42, 43):

(42) $$T_{c}\;\left(K\right)=\;-146.27x_{2}^{2}+346.32x_{2}+308.8$$

(43) $$P_{c}\;\left(kPa\right)=\;-0.8036T_{c}^{2}+660.06T_{c}-119359$$

where *T_c_* is the critical temperature; *P_c_* is the critical pressure, and *x_2_* is the mole fraction of MeOH. The results from the study highlighted the differences and the similarities in the profiles of pressure and density along the columns. It was found that even with the presence of modifiers there was a region closed to the critical isopycnic line, where minimum pressure drop could be achieved. Moreover, the changes in pressure drop were found to be almost insignificant when the operating conditions closed to the isopycnic line.^57^ However, the pressure drop increased more when the trend of operating conditions was perpendicular with isopycnic lines and moved closer to the higher temperature region.

## 4 SUMMARY AND OUTLOOK

pSFC proved itself as a powerful chromatographic separation technique in many research fields. However, the separation mechanism of pSFC has not been fully understood. The dream of “one‐theory‐to‐rule‐chromatographic‐techniques‐all” thus has not yet been fulfilled. Different studies have been done, which contributed valuable understanding for the use of supercritical fluids in practical science. Several approaches to study retention behaviors of pSFC have been applied; each provides parts of a full picture of the separation mechanism. The TM approach contributes to numerous knowledge regarding the changes in the pressure, density, and temperature inside the column. The EM and the ETM show their advantages in constructing the relationship between the analyte retention and several relating factors under pSFC working conditions. However, many conclusions regarding the retention mechanism are different. Mitra concluded that the analyte retention in pSFC was a function of reduced temperature and reduced density (eq. 1, 2), while the LSER approaches concluded that the analyte retention was contributed by a combination of all intermolecular interactions between the analyte molecules and the stationary phases (eq. 35‐39).^114^, ^149^, ^150^, ^151^ To predict the pressure and temperature changes, Kaczmarski's model includes both radial and vertical models (eq. 10‐21), while Leśko's model neglects the radial components (eq. 22‐27).^72^, ^137^ However, each conclusion was made based on different experimental conditions and datasets, hence highlighting their specificities. Therefore, a final statement regarding the retention mechanisms is difficult to make. This also indicates the remaining bottlenecks of pSFC mechanism studies, the lack of a systematic investigation scheme for a global retention mechanism. At the moment, a full guideline for these studies (e.g. criteria for sample sets, suggestion of chromatographic conditions, recommendation of studying approaches and validation strategies, etc.) is still missing. The main reasons slowing down the progress in retention mechanism studies are the complicated combination effects between chromatographic components, the lack of systematic combinations between approaches, the poor experiment designs, and the missing of novel global molecular descriptors. Nevertheless, in near future, other approaches that can account for greater non‐linearity degrees such as the artificial neural networks and the support vector machines may play important roles in the mechanism studies.
